# Love withdrawal predicts electrocortical responses to emotional faces with performance feedback: a follow-up and extension

**DOI:** 10.1186/1471-2202-15-68

**Published:** 2014-06-02

**Authors:** Renske Huffmeijer, Marian J Bakermans-Kranenburg, Lenneke RA Alink, Marinus H van IJzendoorn

**Affiliations:** 1 Centre for Child and Family Studies, Leiden University, P.O. Box 9555, 2300 RB Leiden, the Netherlands; 2 Leiden Institute for Brain and Cognition (LIBC), Leiden University, P.O. Box 9600, 2300 RC Leiden, the Netherlands

**Keywords:** Love withdrawal, Event-related potentials, VPP, LPP, Emotional facial expressions, Performance feedback

## Abstract

**Background:**

Parental use of love withdrawal is thought to affect children’s later psychological functioning because it creates a link between children’s performance and relational consequences. In addition, recent studies have begun to show that experiences of love withdrawal also relate to the neural processing of socio-emotional information relevant to a performance-relational consequence link, and can moderate effects of oxytocin on social information processing and behavior. The current study follows-up on our previous results by attempting to confirm and extend previous findings indicating that experiences of maternal love withdrawal are related to electrocortical responses to emotional faces presented with performance feedback.

**Results:**

More maternal love withdrawal was related to enhanced early processing of facial feedback stimuli (reflected in more positive VPP amplitudes, and confirming previous findings). However, attentional engagement with and processing of the stimuli at a later stage were diminished in those reporting higher maternal love withdrawal (reflected in less positive LPP amplitudes, and diverging from previous findings).

**Conclusions:**

Maternal love withdrawal affects the processing of emotional faces presented with performance feedback differently in different stages of neural processing.

## Background

Parents provide the earliest social environment children come into contact with and, through their parenting efforts and socialization strategies, parents exert a lasting influence on their children’s development, social functioning, and well-being [1,2]. Some socialization strategies, though very effective in the short run, may come at a considerable cost in terms of the child’s later functioning and well-being. Love withdrawal is one such strategy. Love withdrawal is a way of disciplining the child by withholding or withdrawing signs of love and affection in response to the child’s misbehavior or failure. The sense of conditional regard this instils in the child (i.e., the child learns to link compliance and performance to relational consequences) is thought to underlie both the effectiveness and the emotional costs of love withdrawal [3,4] (see also [5,6]). Negative outcomes that have been related to parental, and in particular maternal, use of love withdrawal include feelings of resentment toward the parents, fear of failure, low emotional well-being, and low self-esteem in adolescence and young adulthood [3,4,7-9]. Excessive use of love withdrawal can be considered psychological maltreatment [10].

Much less is known, however, about the effects of love withdrawal on the neural processes that may underlie its behavioral consequences. Knowledge of these neural correlates will aid our understanding of the mechanisms through which parents’ socialization strategies affect socio-emotional development and functioning, and may eventually offer a starting point for parenting support, intervention, and treatment. Recent results from our laboratory have begun to show that experiences of love withdrawal do also relate to the neural processing of socio-emotional information relevant to a link between performance/compliance and relational consequences: Experiences of love withdrawal were related to the amplitude of vertex positive potential (VPP), N400, and late positive potential (LPP) components of the event-related potential (ERP) in response to feedback stimuli combining performance feedback with emotional facial expressions [5,6]. Corroborating evidence comes from a study relating similar neural processes to fear of failure, a characteristic related to love withdrawal [11]. In addition, experiences of love withdrawal have been found to moderate effects of oxytocin on neural indices of social information processing (VPP and LPP, see below) [6], as well as prosocial [12] and altruistic behavior [13,14]. The current study follows-up on our previous work by attempting to confirm and extend previous findings regarding effects of love withdrawal on VPP and LPP amplitudes.

The VPP is a positive deflection in the ERP that peaks at frontocentral electrode sites, roughly between 140 and 180 ms after stimulus onset. The VPP has been associated with the configural processing of faces, showing larger amplitudes in response to emotional compared to neutral expressions [15]. VPP and N170 (that perhaps reflect the opposite side of the same set of generator dipoles [16], but see [17-19]) are often found to be sensitive to intensity, but not valence of emotional expressions [15,20]. The LPP is a centroparietally distributed, positive-going modulation of the ERP beginning about 300–400 ms after stimulus onset [21,22]. The amplitude of the LPP is more positive for emotional stimuli, both pleasant and unpleasant, compared to neutral stimuli. Also, its amplitude may be influenced by both automatic (e.g., capture of attention by unpleasant stimuli) and controlled (e.g., direction of attention away from threatening information and toward non-threatening parts of a stimulus [e.g., a neutral part, such as the sky, on a photograph of a threatening scene]) processes [21-24].

In our previous experiment, which was the first to examine effects of both love withdrawal and oxytocin on event-related potentials (ERPs) to facial feedback stimuli, love withdrawal affected the amplitudes of both these ERP components. ERPs were recorded during performance of a flanker task in response to feedback stimuli combining performance feedback with emotional faces: A picture of a happy or disgusted face was presented in green after each correct response and in red after each error [5,6]. Experiences of maternal love withdrawal both moderated effects of oxytocin on VPP amplitude [6] and predicted more positive VPP amplitudes, indicative of more extensive face processing, under placebo conditions [5]. These results were consistent with the idea that experiences of love withdrawal heighten the relevance of and focus on emotional expressions in performance situations, as these are potential indicators of relational consequences linked to success and failure. More love withdrawal also predicted more positive LPP amplitudes in response to disgusted compared to happy faces, specifically following an erroneous response. This suggested that love withdrawal relates to the allocation of attention toward the motivationally relevant combination of negative feedback with a disgusted face [6].

In light of the recent debate about the (lack of) replicability of findings from psychological research (see e.g., [25] [followed by 15 peer commentaries in the European Journal of Personality]; [26]) it is essential to confirm novel findings, both through replication studies by independent research groups and through follow-up research by the original authors. The current experiment therefore follows-up on our previous work by attempting to confirm and extend the results regarding maternal love withdrawal and ERPs in an independent sample (*n* = 20) using the same facial feedback stimuli described above, but in a slightly adapted experimental context. Because the flanker task, used in our previous experiment, has the disadvantage of eliciting relatively low error rates (causing participants to view more correct than error feedback stimuli), participants in this new experiment performed both the flanker task and a time estimation task. In the latter error rates were manipulated to be approximately 50%. The current study focuses only on love withdrawal (no drugs were administered), and experimental sessions were conducted by experimenters not involved in the previous data collection. We expect that more love withdrawal will be related to more positive VPP amplitudes, as well as to more positive LPP amplitudes in response to disgusted compared to happy faces following erroneous responses.

## Results

### Behavioral data

Participants committed on average 17% errors (*SD* = 7.5%) and responded late on 12% of trials (*SD* = 1.6%) when performing the flanker task. The average error rate on the time-estimation task was 50% (*SD* = 0.6%), and the average reaction time was 1032 ms (*SD* = 43 ms). During the flanker task, participants responded significantly faster to congruent (*M* = 331 ms, *SD* = 24 ms) than to incongruent targets (*M* = 376 ms, *SD* = 28 ms), *t*(19) = 13.56, *p* < .01. Love withdrawal was not significantly correlated with participants’ error percentages and reaction times to congruent and incongruent targets during performance of the flanker task or participants’ reaction times during the time-estimation task (all |*r*s| < .37, all *p*s > .10).

### ERPs

#### VPP

**Figure 1:**
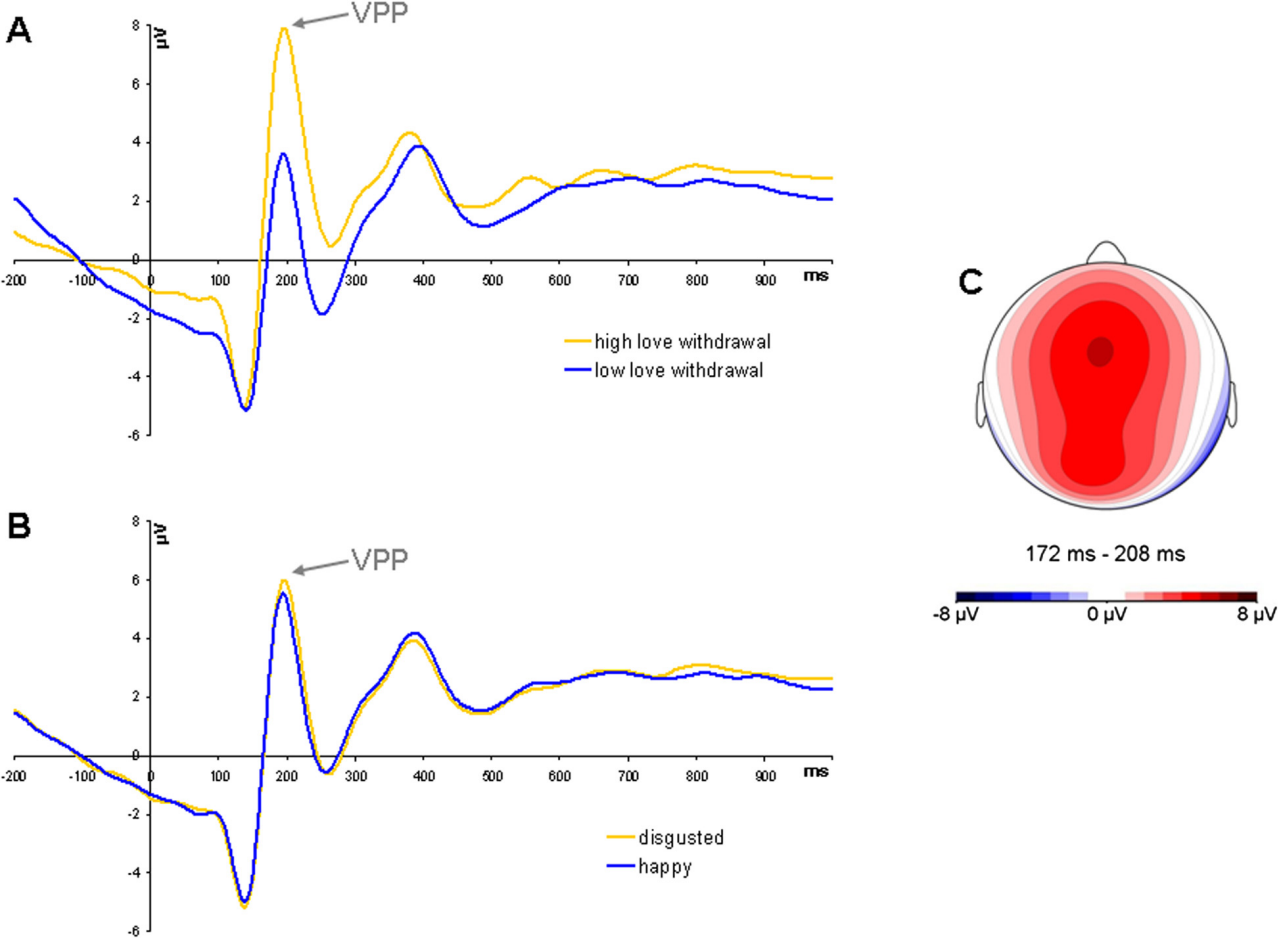
**Grandaverage ERPs at Cz, illustrating the VPP. A**: ERPs to feedback stimuli, averaged across all four categories and across tasks, for participants reporting high and low love withdrawal. Participants were divided into groups for displaying purposes only. **B**: ERPs to happy and disgusted faces, averaged across green and red feedback, and across tasks. **C**: Scalp voltage distribution of the VPP. Participants reporting higher maternal use of love withdrawal showed a more positive response to the feedback stimuli between 170 and 210 ms after stimulus onset (VPP), *F*(1,17) = 11.42, *p* < .01, *η*^*2*^ = .40. More positive amplitudes were also observed in response to disgusted compared to happy faces, *F*(1,17) = 4.73, *p* < .05, *η*^*2*^ = .22. ERPs were low-pass filtered at 15 Hz for displaying purposes only.

The ANCOVA revealed a significant main effect of love withdrawal, *F*(1,17) = 11.42, *p* < .01, *η*^*2*^ = .40. More love withdrawal was related to stronger (more positive) VPP amplitudes (*r* = .59). The main effect of emotion was significant as well, *F*(1,17) = 4.73, *p* < .05, *η*^*2*^ = .22 (reflecting more positive amplitudes to disgusted compared to happy faces; as disgusted and happy faces were not matched for intensity, this may be due to either the valence or the intensity of the facial expressions). The main effect of task (larger amplitudes during performance of the time-estimation task) just failed to reach significance, *F*(1,17) = 4.17, p = .06. In addition, there was a significant 3-way interaction between task, emotion, and love withdrawal, *F*(1,17) = 5.49, *p* < .05, *η*^*2*^ = .25. However, the interaction between emotion and love withdrawal failed to reach significance for both the flanker task and the time-estimation task (*F*(1,17) = 4.35, *p* = .05, *η*^*2*^ = .20 [flanker]; *F*(1,17) = 0.04, *p* > .50 [time-estimation]). No other effects were significant (all *F*s ≤ 3.97, *p*s > .05). Grandaverage ERPs at Cz, illustrating the VPP, are presented in Figure 1.

#### LPP

**Figure 2:**
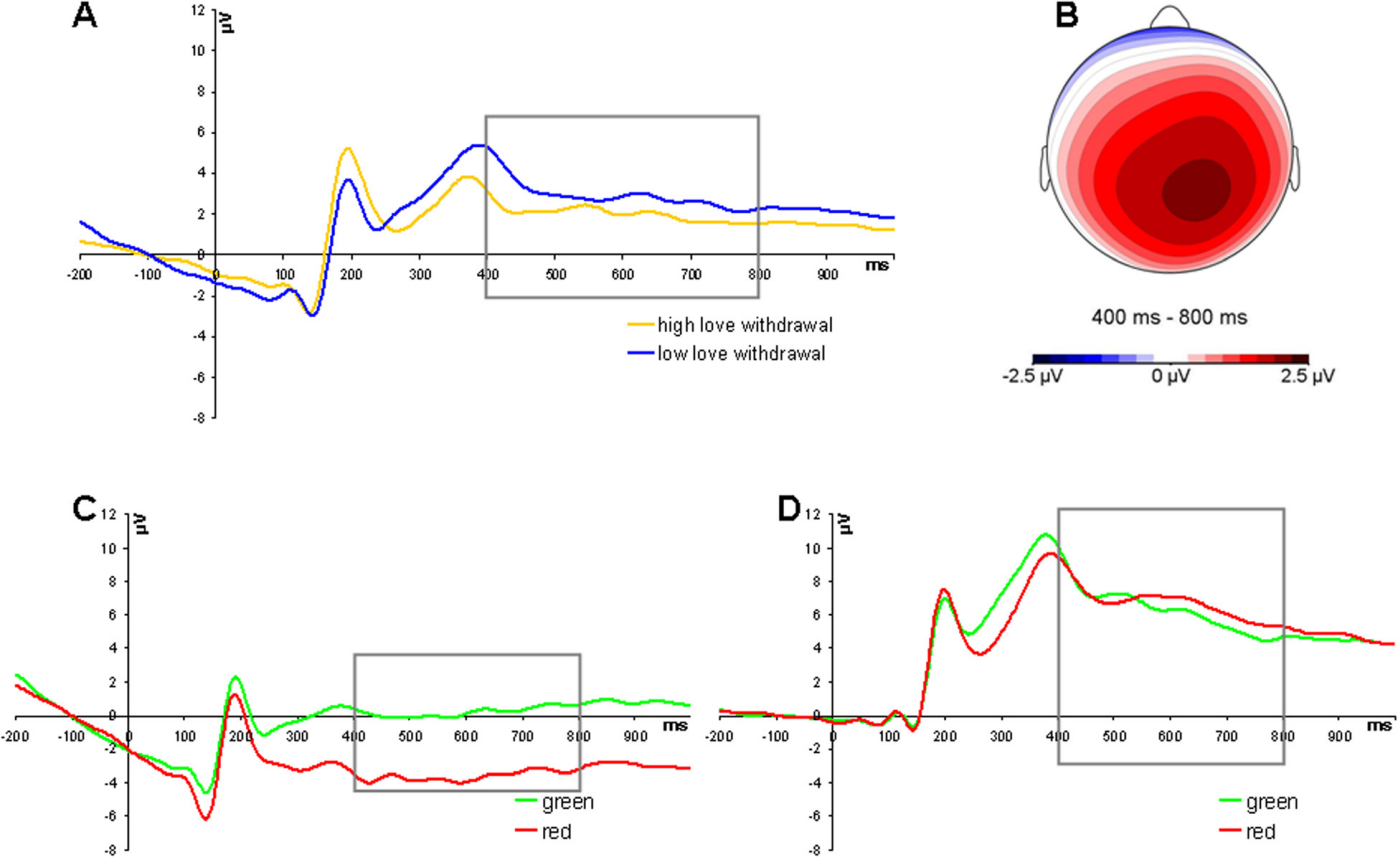
**Grandaverage ERPs at Cz, averaged across electrodes 31, 53, 54, 55 (CPz), 61, 62 (Pz), 78, 79, 80, 86, and Cz, illustrating the LPP. A**: ERPs to feedback stimuli, averaged across all four categories and across tasks, for participants reporting high and low love withdrawal. Participants were divided into groups for displaying purposes only. **B**: Scalp voltage distribution of the color effect on LPP amplitude (green-red). **C**: ERPs to green (correct) and red (error) feedback stimuli recorded during the flanker task. **D**: ERPs to green (correct) and red (error) feedback stimuli recorded during the time-estimation task. Participants reporting higher maternal use of love withdrawal showed a less positive response to the feedback stimuli between 400 and 800 ms after stimulus onset (LPP), *F*(1,17) = 4.65, *p* < .05, *η*^*2*^ = .22. More positive amplitudes were observed during the time-estimation compared to the flanker task, *F*(1,17) = 21.60, *p* < .01, *η*^*2*^ = .56, and in response to green compared to red stimuli, *F*(1,17) = 8.85, *p* < .01, *η*^*2*^ = .34. ERPs were low-pass filtered at 15 Hz for displaying purposes only. The time-window used for analyses of LPP amplitudes is marked with a rectangle.

The ANCOVA revealed a significant main effect of love withdrawal, *F*(1,17) = 4.65, *p* < .05, *η*^*2*^ = .22. More love withdrawal was related to smaller (less positive) LPP amplitudes (*r* = -.48). Significant main effects of task, *F*(1,17) = 21.60, *p* < .01, *η*^*2*^ = .56 (larger LPP amplitudes during performance of the time-estimation task), and color, *F*(1,17) = 8.85, *p* < .01, *η*^*2*^ = .34 (larger LPP amplitudes in response to green compared to red stimuli) were obtained as well. No other main or interaction effects were significant (all *F*s ≤ 3.22, *p*s > .05). Grandaverage ERPs illustrating the LPP are presented in Figure 2.

## Discussion

Maternal use of love withdrawal was significantly related to both VPP and LPP amplitudes in response to the facial feedback stimuli, partially confirming previous results. As expected, and as observed before [5], higher maternal use of love withdrawal was related to larger (more positive) VPP amplitudes. This result is again consistent with the expectation that those who experienced more maternal love withdrawal vigilantly focus on the facial stimuli as potential indicators of the relational consequences linked to failure and success. The finding that participants reporting relatively high maternal use of love withdrawal show heightened processing of emotional faces (presented with performance feedback) thus appears to be robust.

Maternal love withdrawal was also related to LPP amplitudes, but not in the way observed before. Previous results were consistent with an attentional bias toward disgusted faces resulting from an association between performance and relational consequences established through the experience of love withdrawal. In our previous study, higher love withdrawal was related to more positive LPP amplitudes in response to disgusted compared to happy faces, specifically when faces were presented in red, i.e., after an error [6]. However, in the current study higher love withdrawal was related to smaller (less positive) LPP amplitudes, regardless of feedback valence (correct/green or error/red) or the facial emotion displayed (disgusted or happy). Thus, whereas early processing of the facial feedback stimuli was enhanced (reflected in more positive VPP amplitudes), attentional engagement and processing at a later stage seemed to be diminished in those reporting higher maternal use of love withdrawal. Because the current results regarding LPP amplitude did not confirm our previous findings, caution is warranted when interpreting these results. However, it is interesting that a pattern of enhanced early attention to and processing of salient social stimuli, followed by diminished attentional engagement and processing at later stages (i.e., a vigilance-avoidance model) has been suggested to explain attentional biases to threat in persons suffering from (generalized) anxiety disorders (e.g., [27-29]). We speculate that those who have experienced more maternal love withdrawal show, at least in a performance context, a similar vigilance-avoidance response toward emotional faces and/or performance feedback, as these are salient potential indicators of relational consequences linked to failure and success. Alternatively, it might be the case that attentional engagement with and processing of the stimuli at later stages is needed less if early processing is enhanced, i.e., if more or all relevant information has been extracted at earlier stages of processing.

Divergence in the outcomes of our two studies might be related to important differences in study design. In our previous study participants performed the flanker task during both experimental sessions, whereas two different tasks (flanker and time-estimation) were performed during the current study. Although in the current study the effect of love withdrawal did not depend on the task performed, potential influences of task repetition (i.e., performing any task twice) cannot be ruled out. In addition, our previous study focused on effects of oxytocin as well as maternal love withdrawal, and participants were administered oxytocin during one session and a placebo during the other. Effects of love withdrawal on LPP amplitude were not moderated by oxytocin administration in that study [6]. Nevertheless, drug administration was an important component of the experimental context and absent from the current design. Finally, the current study was limited by a small sample size (*n* = 20), limiting the power to detect a complex 3-way interaction between love withdrawal, feedback valence, and emotional expression. As results regarding the LPP did not fully confirm earlier findings, future studies are needed to further elucidate the relation between experiences of maternal love withdrawal and LPP amplitude. In this respect, non-replications can be at least as useful as successful replications of earlier findings and help to move a field forward [30].

Future studies could also address some of the limitations of the current study. First, neutral facial expressions should be included in future experiments to distinguish between the processing of faces in general and facial expressions in particular, which was not possible using our current experimental setup. Furthermore, we measured maternal use of love withdrawal with a self-report questionnaire. There are obvious limitations to the accuracy and reliability of participants’ self-reports. Lastly, our participants were all female. We chose to include only women to maximize the comparability of the current experiment to the one we conducted previously on an independent sample (in which we investigated effects of oxytocin and parental love withdrawal on ERPs in response to facial feedback stimuli [6]). That study included only women, because of the considerable differences between males and females in the oxytocin system [31], and because most of the studies on the behavioral or psychological outcomes of love withdrawal focus on maternal use of love withdrawal with daughters (e.g., [4,8]). Nevertheless, it would be interesting to study the same processes in men. Studies with mixed samples of males and females will facilitate a direct gender comparison, but these samples should be considerably larger to reach sufficient statistical power.

## Conclusions

The current study aimed to contribute to the field by following up on previous findings. The current results partially confirmed previous findings relating experiences of maternal love withdrawal to the processing of emotional faces presented with performance feedback. The results indicated that whereas early processing of these stimuli is enhanced (reflected in more positive VPP amplitudes), attentional engagement and processing at a later stage are diminished in those reporting higher maternal use of love withdrawal.

## Method

### Participants

A total of 26 female undergraduate students, aged 18–24 years (*M* = 19.85, *SD* = 1.85), took part in the ERP experiment that consisted of two sessions separated by approximately four weeks. One participant completed only one session, and data of five other participants could not be analyzed (due to excessive ocular artifacts or due to low error rates on the flanker task leading to insufficient trials for calculation of ERPs in response to red feedback stimuli). The final sample thus consisted of 20 female undergraduate students (age: *M* = 19.85, *SD* = 1.98). They were paid 40 Euros or received course credits for participation. Exclusion criteria were the same as in our previous study, and included colorblindness, smoking, alcohol and drug abuse, neurological and psychiatric disorders, pregnancy, breastfeeding, and use of medication (except oral contraceptives). The study was approved by the ethics committee (“Commissie Medische Ethiek”) of the Leiden University Medical Center (Leiden, the Netherlands).

### Procedure

Participants were asked to come to our laboratory for two experimental sessions, separated by approximately four weeks. Written informed consent for participation was obtained from all participants at the beginning of the first session. Participants were fitted with an electrode net after which they completed either a flanker task or a time-estimation task (with a short break after the fourth block). A random half (*n* = 10) of the participants performed the flanker task during the first session and the time-estimation task during the second session, and the other half performed the time-estimation task during the first session and the flanker task during the second session. At the start of each session, halfway through and after completion of the task participants completed some questionnaires. The questionnaire measuring maternal love withdrawal was administered at the start of the first session.

### Questionnaire

To measure maternal use of love withdrawal, the participants completed the 11-item questionnaire used in our previous experiment ( [5,6]; adapted from the CRPBI and PDQ; [32-35]). Participants rated how well each of the 11 statements described their mother (e.g., “My mother is a person who, when I disappoint her, tells me how sad I make her”) on a 5-point scale ranging from 1 (not at all) to 5 (very well). The average score on the love withdrawal questionnaire was 19.35 (*SD* = 7.91). Both skewness (0.73, *SE* = 0.51) and kurtosis (-0.96, *SE* = 0.99) were acceptable. Cronbach’s alpha was .90 for the current sample. Continuous scores on the love withdrawal questionnaire were used in all analyses.

### Experimental tasks

During each session, participants completed eight 72-trial blocks of a modified Eriksen flanker task [36] or of a time-estimation task, preceded by a 72-trial practice block.

#### Flanker task

Target stimuli consisted of a row of five arrows (7.4° × 1.4° visual angle), presented for 50 ms, all pointing in the same direction (congruent targets), or with the middle arrow pointing in the opposite direction (incongruent targets). Target stimuli were preceded by a fixation cross, presented in black for 1000 ms and then in red for 800–1200 ms (to draw attention to the screen). The participants had to indicate, as fast as possible, whether the middle arrow pointed left or right by pressing the corresponding button on a response pad. To ensure participants would indeed react as fast they could and consequently would commit a substantial number of errors, response deadlines were employed. Because reaction times are generally faster to congruent than to incongruent targets, separate deadlines were used for each target type. New response deadlines were calculated after every block based on the participants’ mean reaction times during that block. Following each response (600–1000 ms after target stimulus offset) a feedback stimulus was presented for 1500 ms. A photograph of a happy or a disgusted face (18.8° × 21.2°) was presented in green if the participant’s response was correct, in red if the participant made an error, and in blue when the participant’s reaction time exceeded the response deadline, resulting in six categories of feedback stimuli: green-happy, green-disgust, red-happy, red-disgust, blue-happy, and blue-disgust. For the current paper, only ERPs in response to green and red stimuli were analyzed because no blue stimuli were presented during the time-estimation task.

#### Time-estimation task

At the beginning of every trial, a fixation cross was presented for 500 ms to draw attention to the screen. Next, a black-and-white picture of a gift box was presented (5.47° × 5.95° visual angle), that changed color (to red-and-yellow) after 1500–2000 ms. The colored picture remained on screen for 2000–2500 ms. Participants’ task was to press a button on a response pad when they thought one second had passed since the picture changed color. For a response to be considered correct (on time) the reaction time needed to be within a margin around 1000 ms. For the first trial of the practice block this margin was set to 1000 +/- 150 ms. The margin was adjusted after every trial. After a correct response 20 ms were taken off the margin (10 ms on each side) and after an incorrect response (both early and late) 20 ms were added to the margin (10 ms on each side). This was done to ensure that all participants committed about 50% errors; it largely eliminated variation in error rates across participants. Participants did not know about the manipulation of error rates. After each response, directly following the offset of the colored picture, a feedback stimulus was presented for 1500 ms. A photograph of either a happy or a disgusted face (18.8° × 21.2°) was presented in green if the participant’s response was correct, and in red if the response was incorrect (early or late), resulting in four categories of feedback stimuli: green-happy, green-disgust, red-happy, and red-disgust.

The same feedback stimuli were used for both the flanker and the time-estimation task (except stimuli were also presented in blue during the flanker task). The same stimuli were also used in our previous study [5,6]. Photographs were selected from Ekman’s [37] standard set of prototypical facial expressions. To make sure the participants would stay involved in the task, they could earn points during the last four blocks of each task. As an incentive toward gaining points the participants were told that the one who had earned the highest number of points by the end of the second session would receive an extra reward.

### ERPs

Participants’ EEG was acquired during task performance using 129-channel hydrocel geodesic sensor nets, amplified using a NetAmps300 amplifier, low-pass filtered at half (i.e., 125 Hz) the digitization rate of 250 Hz, and recorded using NetStation software (Electrical Geodesics, Inc.). Impedances were kept below 50 kΩ. The EEG was high-pass filtered at 0.3 Hz (99.9% pass-band gain, 0.1% stop-band gain, 1.5 Hz roll-off) before exportation for further offline processing using Brain Vision Analyzer 2.0 software (Brain Products). Offline, the EEG was low-pass filtered at 30 Hz (-3 dB, 48 dB/octave) and rereferenced to the average of activity in all channels. Segments extending from 200 ms before to 1000 ms after the onset of each feedback stimulus were extracted, corrected for ocular artifacts using ICA, and averaged per feedback category (green-happy, green-disgust, red-happy, red-disgust) after removal of segments containing residual artifacts (whole segments were removed if the difference between the maximum and minimum activity exceeded 60 μV in the vertical EOG channels [channel 8-channel 126 and channel 25-channel 127] or 40 μV in the horizontal EOG channel [channel 128-channel 125], and individual channels were removed from a segment if the difference between the maximum and minimum activity in that channel during that segment exceeded 150 μV).

On average, participants contributed 451 artifact-free trials during performance of the flanker task (*M* = 186, *SD* = 29 [green-happy]; *M* = 184, *SD* = 27 [green-disgust]; *M* = 40, *SD* = 21 [red-happy]; *M* = 41, *SD* = 21 [red-disgust]) and 507 during performance of the time-estimation task (*M* = 129, *SD* = 22 [green-happy]; *M* = 127, *SD* = 21 [green-disgust]; *M* = 126, *SD* = 22 [red-happy]; *M* = 125, *SD* = 18 [red-disgust]). There were no significant differences between the flanker and time-estimation task in the percentage of artifact-free trials for any feedback category (all *t*s ≤ 1.21, *p*s > .10). For each of the resulting ERPs a 200 ms pre-stimulus baseline was subtracted from all data points.

Time windows and electrodes for quantification of the VPP and LPP were chosen based on a-priori considerations verified by visual inspection of the raw ERP waveforms and difference waves. The VPP is known to peak at frontocentral electrode sites and is often quantified at the vertex electrode (see e.g., [19]). Visual inspection of the ERPs time-locked to the onset of the feedback stimuli revealed a clear positive peak, maximal close to Cz at approximately 190 ms after feedback onset. The VPP was therefore defined as the average amplitude in the 170–210 ms post-stimulus interval at electrode Cz.

The LPP is a modulation of ERP amplitude at centroparietal electrode sites, visible as a difference between the ERPs to different classes of stimuli, rather than a peak in the raw ERP waveform. This positive-going modulation of ERP amplitude begins approximately 300–400 ms after feedback onset and lasts for several hundreds of milliseconds [21,22]. In particular in ERPs recorded during the flanker task, visual inspection revealed such a modulation in the ERPs in response to green compared to red stimuli. This modulation started between 300 and 400 ms after stimulus onset and lasted throughout the ERP. The component looked highly similar to what we observed previously [6]. The LPP was therefore defined as the average amplitude in the 400–800 ms post-stimulus interval averaged across 11 centroparietal electrodes (31, 53, 54, 55 [CPz], 61, 62 [Pz], 78, 79, 80, 86, and Cz).

### Analyses

Statistical analyses were performed using SPSS 19 software. To test the hypotheses, repeated measures ANCOVAs were performed with VPP and LPP amplitudes as dependent variables, task (flanker vs. time-estimation), color (green vs. red), and emotion (happy vs. disgusted) as within-subjects factors, order of task performance (flanker first vs. time-estimation first) as between-subjects factor, and love withdrawal as continuous predictor.

## Competing interests

The authors declare that they have no competing interests.

## Authors’ contributions

All authors (RH, MJBK, LRAA, and MHvIJ) were extensively involved in the design of the study and contributed to data-analysis. RH coordinated data collection and wrote a first draft of the manuscript. All authors contributed substantially to editing and refining the final draft of the manuscript. All authors have read and approved the manuscript.
